# An examination of nervous system revealed unexpected immunoreactivity of both secretory apparatus and excretory canals in plerocercoids of two broad tapeworms (Cestoda: Diphyllobothriidea)

**DOI:** 10.1017/S0031182023000306

**Published:** 2023-06

**Authors:** Daniel Barčák, Anna Alexovič Matiašová, Eva Čisovská Bazsalovicsová, Miroslava Soldánová, Mikuláš Oros, Ivica Králová-Hromadová

**Affiliations:** 1 Institute of Parasitology, Slovak Academy of Sciences, Košice, Slovak Republic; 2 Faculty of Science, Institute of Biology and Ecology, P.J. Šafárik University in Košice, Slovak Republic; 3 Institute of Parasitology, Biology Centre, Czech Academy of Sciences, České Budějovice, Czech Republic

**Keywords:** CLSM, *Dibothriocephalus*, *Diphyllobothrium*, fish-borne parasitic disease, frontal glands, zoonosis

## Abstract

*Dibothriocephalus ditremus* and *Dibothriocephalus latus* are diphyllobothriidean tapeworms autochthonous to Europe. Their larval stages (plerocercoids) may seriously alter health of their intermediate fish hosts (*D. ditremus*) or cause intestinal diphyllobothriosis of the final human host (*D. latus*). Despite numerous data on the internal structure of broad tapeworms, many aspects of the morphology and physiology related to host–parasite co-existence remain unclear for these 2 species. The main objective of this work was to elucidate functional morphology of the frontal part (scolex) of plerocercoids, which is crucial for their establishment in fish tissues and for an early attachment in final hosts. The whole-mount specimens were labelled with different antibodies and examined by confocal microscope to capture their complex 3-dimensional microanatomy. Both species exhibited similar general pattern of immunofluorescent signal, although some differences were observed. In the nervous system, FMRF amide-like immunoreactivity (IR) occurred in the bi-lobed brain, 2 main nerve cords and surrounding nerve plexuses. Differences between the species were found in the structure of the brain commissures and the size of the sensilla. Synapsin IR examined in *D. ditremus* occurred mainly around FMRF amide-like IR brain lobes and main cords. The unexpected finding was an occurrence of FMRF amide-like IR in terminal reservoirs of secretory gland ducts and excretory canals, which has not been observed previously in any tapeworm species. This may indicate that secretory/excretory products, which play a key role in host–parasite relationships, are likely to contain FMRF amide-related peptide/s.

## Graphical abstract

**Figure:**
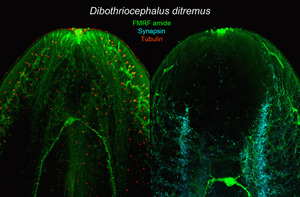

## Introduction

The broad tapeworms (the order Diphyllobothriidea) are parasites of fish-eating mammals and birds, which became infected by ingestion of plerocercoids (second larval stage) in a fish prey. The order currently harbours about 70 species (Kuchta and Scholz, 2017), some of which pose a serious threat to their fish intermediate hosts (Bylund and Andersen, 1994; Rahkonen *et al*., 1996). Other species cause human diphyllobothriosis, an intestinal disease with millions of cases worldwide (Chai *et al*., 2005). Two autochthonous European species of recently resurrected genus *Dibothriocephalus* (Lühe, 1899) (see Waeschenbach *et al*., 2017) were chosen as models for this study with respect to the different pathogenic effects on their hosts. *Dibothriocephalus ditremus* (Creplin, 1825) is widespread in Circumboreal region, and its most frequent second intermediate hosts are salmonid and coregonid fishes (see Halvorsen, 1970; Valtonen and Julkunen, 1995). This tapeworm acts as an important ecological driver of population dynamics of wild salmonids (Halvorsen and Andersen, 1984), and heavy infections caused multiple organ damage in farmed salmons and trouts (Weiland and Meyers, 1989; Rodger, 1991; Rahkonen *et al*., 1996). Human broad tapeworm, *Dibothriocephalus latus* (Linnaeus, 1758), is the most common causative agent of human diphyllobothriosis in Europe, where it currently persists mainly in Russia and in the Alpine lakes region (Králová-Hromadová *et al*., 2021). Plerocercoids are often localized in the muscles of fish hosts and consumption of raw dishes made from European perch (*Perca fluviatilis*) and Northern pike (*Esox lucius*) is the most important way of the parasite transmission into human hosts (Gustinelli *et al*., 2016). The pathological effect of larvae and adults is mainly determined by mechanical damage of host tissues and activity of chemical substances released into host body. An important role is played by the scolex (head of the tapeworm), which is equipped with 2 specialized muscular attachment organs (bothria), products of secretory apparatus (frontal glands) are released on its tip (Öhman-James, 1973) and both bothrial musculature and secretory cell apparatus are closely associated with primitive brain in its anterior part (Gustafsson and Vaihela, 1981; Halton and Maule, 2004; Biserova and Kemaeva, 2012). The nervous and secretory systems can hardly be visualized by conventional histological techniques, and therefore, immunohistochemical methods and immunofluorescent dyes are used in combination with confocal microscopy to study their anatomy and chemical nature. Among diphyllobothriideans, *Dibothriocephalus dendriticus* (Nitzsch, 1824) has often served as a model species for such studies (e.g. Öhman-James, 1968; Gustafsson *et al*., 1986; Gustafsson and Wikgren, 1989; Lindholm *et al*., 1998; Biserova and Kutyrev, 2014; Kutyrev *et al*., 2017), while only few data are available for *D. ditremus* and *D. latus* (Öhman-James, 1973; Barčák *et al*., 2019).

The nervous system of flatworms contains a variety of neuroactive substances including neuropeptides (a diverse group usually consisting of 3–40 amino acid residues), which were recognized as neuromodulators, neurotransmitters or hormones in both invertebrates and vertebrates (Jekely, 2013). Their functional features are often determined by amidation of their carboxy terminal (Bradbury and Smyth, 1991) and some of them were investigated as potential targets for novel anthelminthic drugs (Maule *et al*., 2002). Specific antibodies against one of these amidated neuropeptides, a tetrapeptide FMRF amide (Phe-Met-Arg-Phe-NH_2_), were successfully applied as a neuronal immunohistochemical marker in many invertebrate taxa including parasitic flatworms (Fairweather and Halton, 1991). However, the peptide itself has never been confirmed in any flatworm taxa, and therefore, anti-FMRF amide antibodies obviously cross-react with structurally similar (FMRF amide-like) peptides of flatworms (Lange *et al*., 1991). The complexity of the nervous system calls for an application of several different markers. An antibody SYNORF1 raised against a phosphoprotein synapsin, a regulator of neurotransmitter vesicles release from presynaptic membranes (Hilfiker *et al*., 1998; Kao *et al*., 1999), was previously used as a pan-neuronal marker in both planarian and tapeworm species (Cebrià, 2008; Rozario and Newmark, 2015). Anti-tubulin antibodies have been applied for visualization of microtubules in nervous fibres, walls of frontal gland ducts or flame cells of excretory system in our previous study on *D. latus* (Barčák *et al*., 2019) as well as in other studies (e.g. Moreno *et al*., 2001; Rozario and Newmark, 2015; Kutyrev *et al*., 2017).

The function of the secretory apparatus (frontal glands) in tapeworms has not yet been clearly explained. In general, the secretion might facilitate penetration of the plerocercoids through the host's intestinal wall, migration into its musculature and early adhesion in the intestine of final host (Kuhlow, 1953; Öhman-James, 1973; Kuperman and Davydov, 1981). The ducts of secretory cells run towards the apical pit in the scolex, where secretory granules are stored in the reservoirs, and released to host tissues (Öhman-James, 1973; Kuperman and Davydov, 1981). The ultrastructure of tapeworm secretory cells suggested their capability of protein synthesis (Richards and Arme, 1981; Barčák *et al*., 2019); however, secretory granules inside the ducts are generally unreactive to cytochemical tests (Öhman-James, 1973). In the previous study on *D. latus*, *β* tubulin immunoreactive (IR) reservoirs of putative secretory glands around apical pit were tagged, but chemical composition of their content remained unknown (Barčák *et al*., 2019). The excretory system of tapeworms, which is not yet fully understood, has an osmoregulatory and/or excretory function performed by protonephridian flame cells, from which ‘waste’ products are released through a system of canals terminated at the end of the body (Webster and Wilson, 1970; Lumsden and Hildreth, 1983; Mustafina and Biserova, 2022). Recent data suggested a functional complexity of the excretory system of diphyllobothriideans, because an immunomodulator prostaglandin E_2_ was detected in the flame cells (Kutyrev *et al*., 2017). The products of glandular apparatus and excretory system represent a major part of a complex and poorly explored mixture of various molecules called secretory/excretory products, which have prominent place in host–parasite relationships and are studied as potential diagnostic markers and immunomodulatory drugs (e.g. Lightowlers and Rickard, 1988; Harnett, 2014).

The primary aim of the present study was to investigate the functional morphology of infectious larvae (plerocercoids) of 2 broad tapeworms, *D. ditremus* and *D. latus*. The novelty of this work was application of multi-marker immunofluorescent labelling and confocal microscopy on whole-mount specimens, in order to capture complex 3-dimensional structure of the scolex. The cross-reactivity of anti-FMRF amide marker is briefly discussed with regards to genome-based prediction of FaRPs (also known as FaLPs, FMRF amide-like peptides) in *D. latus.*

## Materials and methods

The plerocercoids of *D. ditremus* were isolated from cysts located in the body cavity of brown trout *Salmo trutta* m. *fario* from Lake Takvatn (69°6′37″N, 19°4′29″E) and Lake Kalandsvatn (60°16′19″N, 5°23′55″E) in Norway, sampled in October 2017 and August 2018, respectively. The plerocercoids of *D*. *latus* were isolated from the musculature of European perch *P. fluviatilis* from Lake Iseo (45°44′20″N, 10°4′15″E) in Italy, sampled in April 2018. After the isolation, the larvae of both species were rinsed in 0.1 M phosphate-buffered saline (PBS) buffer, immediately heat-treated by almost boiling PBS for about 1 min, fixed in freshly prepared 4% paraformaldehyde solution and stored in PBS with 0.03% sodium azide at 4°C (details in Barčák *et al*., 2019). Several specimens of *D. ditremus* were fixed using standard procedure, i.e. heat-treatment in PBS was omitted. These plerocercoids were used for test of anti-FMRF amide primary antibody's specificity (see below).

The immunofluorescence labelling of 12 and 4 plerocercoids of *D. ditremus* and *D. latus*, respectively, was initiated by rinsing in PBS, which was followed by permeabilization by 0.5% Triton X-100 in PBS (PBSTrX). The non-specific signal was blocked by the incubation of samples in 5% normal donkey serum (NDS; #ab10352, Abcam, Cambridge, UK) in PBSTrX for 2 h. The immunolabelling was performed using polyclonal rabbit anti-FMRF amide IgG antibody (#ab10352, Abcam; diluted 1:1000), monoclonal mouse anti-SYNORF1 (anti-synapsin) IgG2b antibody (clone 3C11, DSHB, Iowa, USA; diluted 1:10) and monoclonal mouse anti-*α* tubulin and anti-*β* tubulin IgG1 antibodies (clones 12G10 and E7 – applied individually or as a mixture, both DSHB; diluted 1:10 each) in 5% NDS in PBSTrX for 7 days at 4°C. The unbounded primary antibodies were rinsed off by 3 baths in PBSTrX, while tagged structures were visualized by secondary antibodies donkey anti-rabbit Alexa Fluor 488 and donkey anti-mouse Alexa Fluor 555 (#A-21206 and A-31570; both Thermo Fisher Scientific, Waltham, USA; diluted 1:500) in 5% NDS in PBSTrX for 2 days at 4°C. After rinsing in PBS, the specimens were incubated in nuclear stain Draq5 (#4084, Cell Signalling Technology, Danvers, MA, USA; diluted 1:500) in PBS for 5 min, rinsed and mounted in antifade reagent ProLong® Gold (#9071, CST). Additionally, 4 heat-treated specimens of *D. ditremus* were used as the negative control, i.e. the same protocol was used for their labelling, but the primary antibodies were omitted. No specific signal was observed for both secondary antibodies (Supplementary file S1). Eight conventionally fixed plerocercoids of *D. ditremus* were labelled using the same protocol as was used for heat-treated specimens to detect possible false-positive results. As FMRF amide-like IR was identical on both types of samples, the signal on heat-treated specimens is considered specific. It is worthy to mention that heat-treatment caused ‘natural’ relaxation of tapeworm bodies and thus decreased its thickness making it easier to transilluminate in the confocal microscope.

The optical scans were captured by confocal microscope Leica TCS SP5 X equipped with software LAS AF (Leica Microsystems, Mannheim, Germany) and processed as maximum intensity Z-stack projections of multiple optical sections in graphic platform FIJI (NIH, USA) (Schindelin *et al*., 2012). The terminology of nervous system follows Richter *et al*. (2010).

Considering lack of original FMRF amide in flatworms and immunoreactivity of anti-FMRF amide antibodies with FMRF amide-related peptides, structures labelled with this antibody will herein be termed as FMRF amide-like immunoreactive (IR) rather than FMRF amide IR (see also Richter *et al*., 2010). Both anti-*α* tubulin and anti-*β* tubulin antibodies were applied individually; however, better results were gained when they were used as the mixture.

## Results

### Immunofluorescence labelling of *Dibothriocephalus ditremus*

**Fig. 1.**
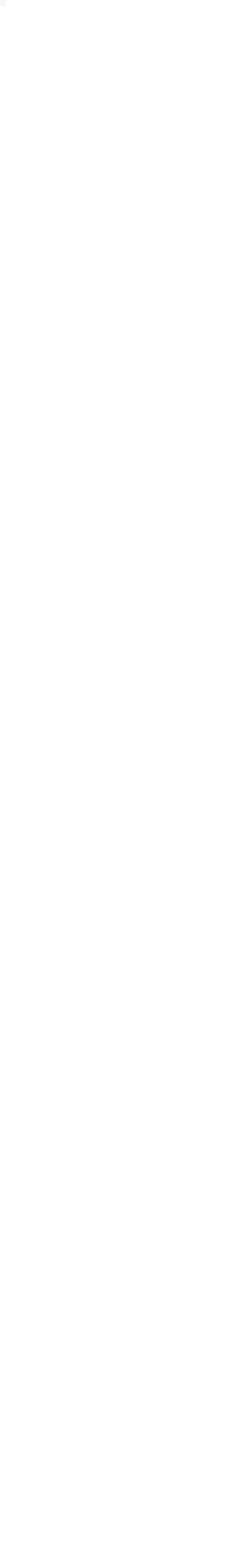
Plerocercoids of *Dibothriocephalus ditremus* with the FMRF amide-like and tubulin IR structures, overall view and nervous system. (A–C, F–I) Dorso-ventral views; (D, E) lateral views. (A) Microanatomy of anterior part of scolex in general, note bi-lobed brain, two main nerve cords, ducts of secretory cells with their reservoirs and excretory system with cortical network of canals and flame cells; merged. (B) Bi-lobed brain with thin interlobar neurites, commissural neurons and large neuron and its two dominant neurites; merged. (C) Large neuron with its two dominant neurites, and additional thin neurites; left picture merged, right Draq 5. (D) Lobe of brain split into dorsal and ventral part on level of apical pit, note also neuron somas (arrowheads) in lobe; merged. (E) Lobe of brain joined with main cord and nerve plexus, note also sub-tegumental plexus, neuron bodies (arrowheads) and interconnecting neurites (same field as 1D, but lateral instead of medial layers were selected for Z-stack); merged. (F) Neurites of receptor cells under tegument in apical area and neuron somas (arrowheads), merged. (G) FMRF amide-like IR fibre-like sensilla (arrow) of receptor cell neurites that penetrate into tegument, merged. (H) FMRF amide-like IR bulb-like sensilla (arrow) of receptor cell neurite embedded in tegument; merged. (I) FMRF amide-like neurite of receptor cell with flatten sensilla (arrow) near basal lamina of tegument; merged. (J) FMRF amide-like IR fibre-like sensilla (arrow) of receptor cell neurites that penetrate into tegument on margin of bothrium; left picture merged, middle FMRF amide-like IR, right tubulin IR. (K) Tubulin IR sensilla (arrows) of the receptor cells embedded in tegument near apical pit; left picture merged, middle FMRF amide-like IR, right tubulin IR. AP, apical pit; CN, commissural neuron; DS, ducts of secretory cells; EC, excretory canal; FC, flame cell; LB, lobe of brain; LCN, large commissural neuron; MNC, main nerve cord; N, nucleus; NP, nerve plexus; NT, neurite; RSD, reservoir of secretory cell duct; RN, neurite of receptor cell; SB, surface of bothrium; SP, sub-tegumental plexus; T, tegument.

**Fig. 2.**
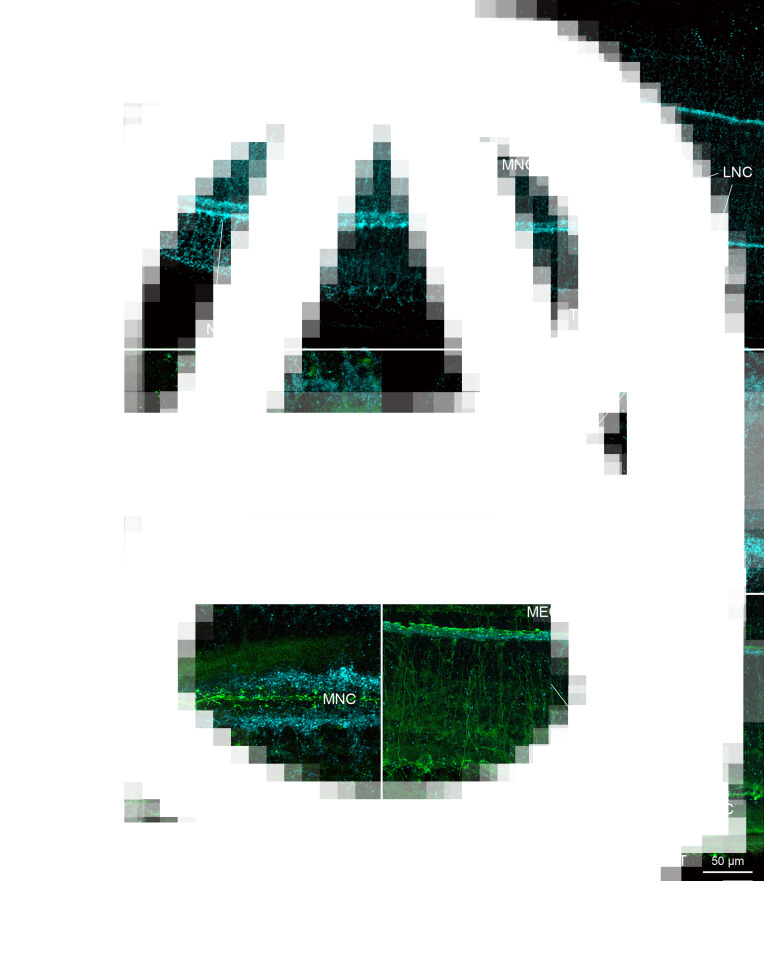
Plerocercoids of *Dibothriocephalus ditremus* with the synapsin IR and FMRF amide-like IR structures of nervous system, and FMRF amide-like IR main excretory canals. Dorso-ventral views. (A) Scolex and neck with synapsin IR nervous system, positions of large commissural neuron and posterior end of bothria marked with asterisk and dashed line, respectively. (B) Anterior part of scolex with lobe of brain, and brain commissure with neurons; left picture merged, right picture synapsin IR. (C) Middle part of scolex with lobe of brain, large commissural neuron and medullar nerve cord associated with nerve plexus; left picture merged, right picture synapsin IR. (D) Anterior part of neck with main nerve cord with adjacent neurites and main excretory canal; left picture merged, middle picture FMRF amide-like IR, right picture synapsin IR. AP, apical pit; CN, commissural neuron; EC, excretory canal in cortical parenchyma; LB, lobe of brain; LCN, large commissural neuron; MEC, main excretory canal; MNC, main nerve cord; NB, nerve bundle; NP, nerve plexus; NT, neurite; T, tegument.

The nervous system of *D. ditremus* exhibited FMRF amide-like IR in the bi-lobed brain, 2 main nerve cords, nerve plexus formed around the main cords, sub-tegumental nerve plexus, 3 types of receptor cells and interconnecting neurites (Figs 1A–J and 2A–C). The commissural connection of the brain possessed thin neurites, and commissural neurons (6–9 *μ*m in diameter, *n* = 5), including single large intensively FMRF amide-like IR neuron (Figs 1A–C and 2B and C). This large neuron (11–17 *μ*m in diameter, *n* = 6) released 2 dominant neurites, running through the main nerve cords towards posterior part of the body, as well as several thin neurites (Figs 1A–C, 2B and C). Two lobes of the brain, interconnected by the commissure, possessed a dense net of FMRF amide-like IR neurons and their neurites (Fig. 1B). Anteriorly, each lobe was split into dorsal and ventral part, while posteriorly these 2 parts were fused and connected with the main nerve cord. Additionally, the lobe communicated also with the nerve plexus around the main cord (Fig. 1D and E). Each main nerve cord released thin neurites towards the nerve plexus, which surrounded the cord dorsally, ventrally and laterally; thin neurites also run towards body surface and form FMRF amide-like IR sub-tegumental nerve plexus (Fig. 1E).

Four types of receptor cells were identified in the apical part of the scolex, based both on the morphology of their terminals (sensilla) embedded in the tegument and on their immunoreactivity. The sensilla, which were both FMRF amide-like IR and tubulin IR, were ended with (i) a simple fibre (fibre-like type) of approximately same width as a neurite itself (Fig. 1F and G), (ii) a bulb-like enlargement (width 1.27–1.55 *μ*m, *n* = 3) embedded in the distal cytoplasm of the tegument (Fig. 1H) or (iii) a flatten structure (width 1.1 *μ*m, *n* = 1) situated just under the basal lamina of the tegument (Fig. 1I). Fibre-like sensilla were the only FMRF amide-like IR type observed on the edge of bothria (Fig. 1J). The fourth type consisted exclusively of tubulin IR sensilla of bulb-like shape (width 1.2–1.7 *μ*m, *n* = 11), which occurred in the apical part of the scolex. In general, the tubulin IR sensilla were much more numerous than the FMRF amide-like IR sensilla (Fig. 1K).

The synapsin IR was observed mainly in the area of brain lobes and main nerve cords as an aggregation of a point-like signal, which predominantly surrounded these structures (Fig. 2A). A less intensive synapsin IR occurred in both the neurites released from the brain lobes towards the apical pit and in the neurite bundles, which left the lobes and the cords (Fig. 2B and C). The bundles run towards the body surface, where they joined the neuron plexuses (Fig. 2C). In the anterior part of the neck, synapsin IR was observed in the proximity of FMRF amide-like IR main nerve cords, from which numerous FMRF amide-like IR neurites run laterally, but only few of them exhibited co-localization with synapsin IR (Fig. 2D). Longitudinal and transversal minor nerve cords in the neck also showed synapsin IR (Fig. 2A).

**Fig. 3.**
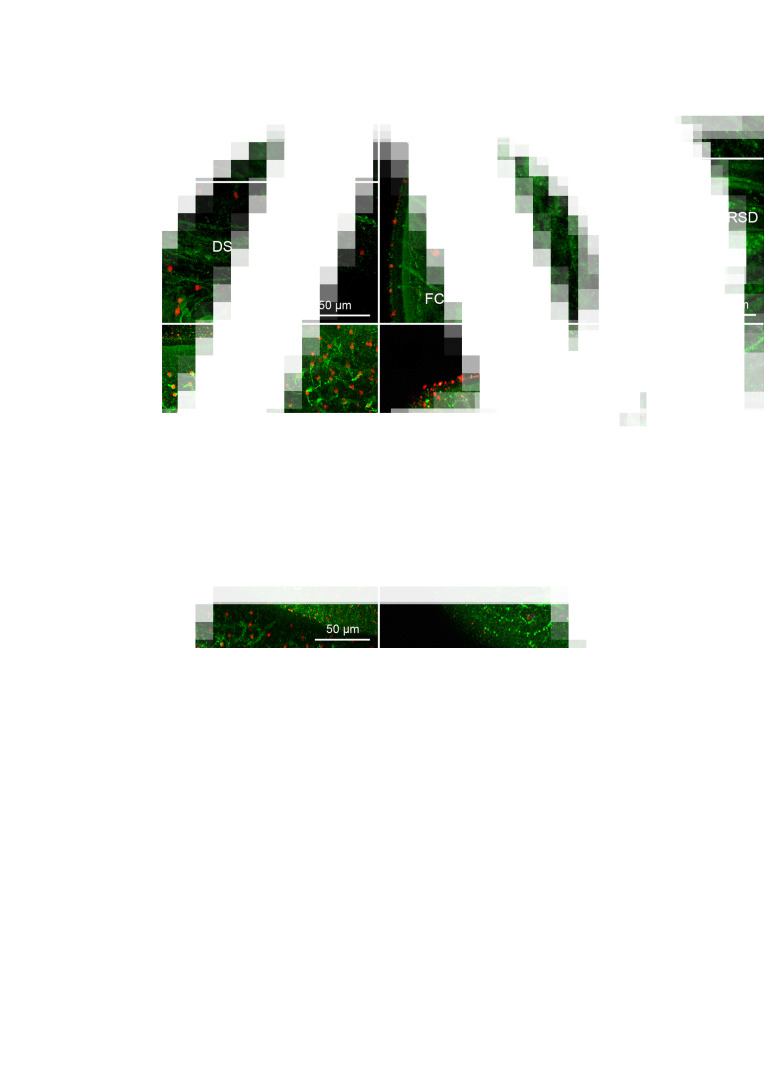
Plerocercoids of *Dibothriocephalus ditremus* with the FMRF amide-like and tubulin IR structures of secretory cell apparatus and excretory system. (A–F) Dorso-ventral views, (G) lateral view. All merged. (A) Anterior part of scolex with secretory cell ducts that run towards apical area of scolex and cortical excretory canals connected with flame cells. (B) Secretory cell reservoirs, which open into apical pit. (C) Anterior part of scolex with numerous secretory cell ducts, which open in proximity of apical pit. (D) Sub-tegumental part of secretory cell reservoirs with their granular content and connections with environment. (E) An anterior part of scolex with cortical network of excretory canals and numerous flame cells. (F) Three flame cells each possessing single flame flagellum and their connection with cortical excretory canal *via* collecting ducts. (G) Anterior part of the scolex with cortical network of excretory canals associated with cell somas (arrowheads). AP, apical pit; CD, collecting duct of excretory system; DS, ducts of secretory cells; EB, edge of bothrium; EC, excretory canal; FC, flame cell; FF, flame flagellum; LB, lobe of brain; LNC, longitudinal minor nerve cord; MNC, main nerve cord; RSD, reservoir of secretory cell duct; T, tegument; TNC, transversal minor nerve cords.

The secretory apparatus of *D. ditremus* also exhibited FMRF amide-like IR. The ducts of secretory cells were observed running laterally to the brain–main nerve cord complex towards an apical area of the scolex (Figs 1A and 3A). The ducts were terminated with large sub-tegumental reservoirs (up to 10 *μ*m in diameter) with granular content (Fig. 3A–D), which was released to the environment through thin tegument-penetrating canals (Fig. 3B and D). Majority of the secretory cell ducts were terminated in the apical pit or in its proximity, while a relatively low number penetrated the tegument more posteriorly (Fig. 3A and C).

The excretory system showed both FMRF amide-like IR and tubulin IR (Fig. 3E–G). The FMRF amide-like IR was observed in tubular structures, i.e. in a network of excretory canals mostly situated in the cortical parenchyma and associated with cell somas (Fig. 3E and G). These canals released collecting ducts, whose funnel-like terminals surrounded tubulin IR flame flagellum (Fig. 3F). These initial structures represented the excretory functional unit – flame cell, also known as protonefridium. In the neck, a pair of FMRF amide-like IR main excretory canals was observed (Fig. 2C).

### Immunofluorescence labelling of *Dibothriocephalus latus*

**Fig. 4.**
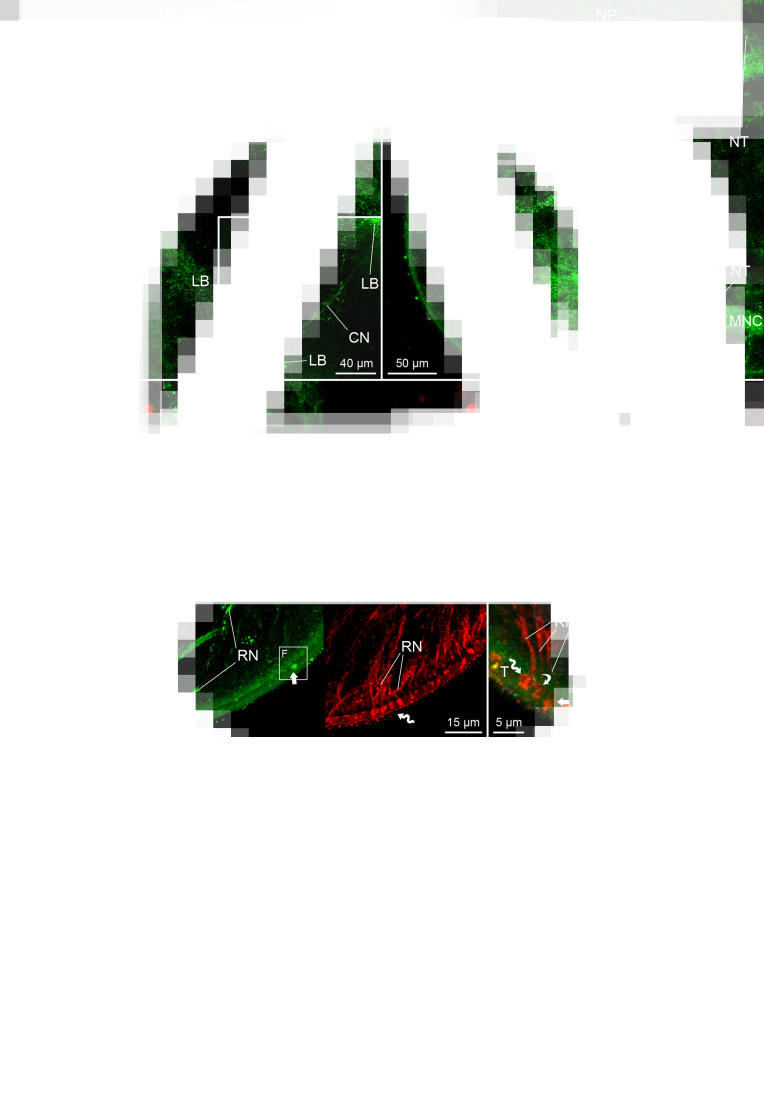
Plerocercoids of *Dibothriocephalus latus* with the FMRF amide-like and tubulin IR structures, overall view and nervous system. (A–G) Dorso-ventral views. (A) Microanatomy of anterior part of scolex in general, note lobes of brain, terminal parts of secretory cell ducts near apical pit and cortical excretory canals with flame cells; merged. (B) Commissure of brain with commissural neuron, same specimen as in A; FMRF amide-like IR. (C) Scolex with lobes of brain, commissural neurons, one of which is intensively FMRF amide-like IR and nerve plexuses interconnected with main nerve cords by thin neurites; FMRF amide-like IR. (D) Anterior part of brain lobe with neuron somas (arrowheads), note FMRF amide-like IR neurite released from lobe possessed flatten type of sensillum (curved arrow) situated in basal lamina of tegument, and FMRF amide-like IR bulb-like sensillum (arrow) embedded in tegument, and numerous tubulin IR neurites of receptor cells with bulb-like sensilla (sinuous arrow); left picture merged, middle FMRF amide-like IR, right tubulin IR. (E) FMRF amide-like IR fibre-like type of sensillum (arrow), which penetrate tegument, and its neurite; left picture merged, middle FMRF amide-like IR, right tubulin IR. (F) FMRF amide-like IR bulb-like sensillum (arrow) embedded in tegument and its neurite; (G) Tubulin IR bulb-like sensilla with internal cilium (sinuous arrow), external cilium (arrow) and lack of cilium (curved arrow) and their neurites; left picture merged, middle FMRF amide-like IR, right tubulin IR. AP, apical pit; CN, commissural neuron; EC, excretory canal; FC, flame cell; LB, lobe of brain; MNC, main nerve cord; NP, nerve plexus; NT, neurite; RN, neurite of receptor cell; RSD, reservoir of secretory cell duct; SP, sub-tegumental plexus; T, tegument.

The nervous system of *D. latus* exhibited FMRF amide-like IR in the brain, main nerve cords, nerve plexus and neurites around the cords, and in some neurites of receptor cells (Fig. 4A–F). The bi-lobed brain possessed several FMRF amide-like IR commissural neurons, one of which exhibited more intensive signal and larger dimensions (9–13 *μ*m in diameter, *n* = 3) than the others (7–10 *μ*m, *n* = 10) (Fig. 4B and C). The lobes of the brain possessed FMRF amide-like IR neuron bodies and neurites (Fig. 4C and D), the latter ran through the main nerve cords towards posterior end of the body. From an anterior part of the lobe, an FMRF amide-like IR neurite of receptor cell ran towards the apical area of the scolex (Fig. 4D).

Four types of sensilla of the receptor cells were observed in *D. latus*. The FMRF amide-like IR types could be additionally distinguished on the basis of their morphology and localization as (i) a thin fibre-like type, which penetrated the tegument (Fig. 4E), a bulb-like type (width of the bulb 2.4–3.2 *μ*m, *n* = 5) that was embedded in the distal cytoplasm (Fig. 4D and F) or a flatten type (width 2.4–3.4 *μ*m, *n* = 3) situated beneath the basal lamina (Fig. 4D). Their neurites usually exhibited co-localization of FMRF amide-like IR and tubulin IR (Fig. 4D–F). However, a large number of the sensilla and their neurites were solely tubulin IR (Fig. 4D). This fourth type occurred in the apical part of the scolex and their bulbs (width 1.6–2.8 *μ*m, *n* = 14) were embedded in the tegument. The solely tubulin IR sensilla were not uniform considering their morphology, because some of them possessed a central cilium, which was inside their bulb, while in others, the cilium was protruded or it was not observed at all (Fig. 4G).

**Fig. 5.**
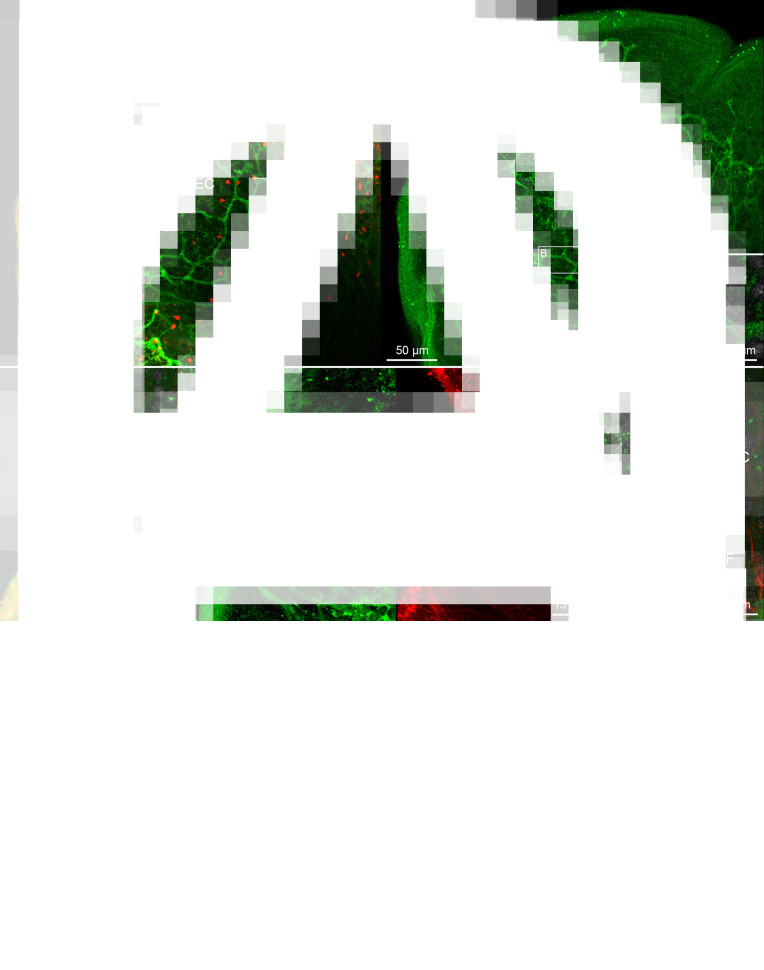
Plerocercoid of *Dibothriocephalus latus* with the FMRF amide-like and tubulin IR structures of both secretory cell apparatus and excretory system. (A–D) Dorso-ventral view. (A) Anterior part of scolex with reservoirs of secretory cell ducts near the apical pit, and network of cortical excretory canals and numerous flame cells; left picture merged, right FMRF amide-like IR. (B) Detail of excretory canal and the nucleus (arrowhead); merged picture. (C) Reservoirs of secretory cell ducts under tegument in apical pit; left picture merged, middle FMRF amide-like IR, right tubulin IR. (D) Connection of flame cell possessing tubulin IR flame flagellum with FMRF amide-like IR excretory canal by collecting duct, merged. AP, apical pit; CD, collecting duct; DS, duct of secretory cell; EC, excretory canal; FC, flame cell; FF, flame flagellum; N, nucleus; RSD, reservoir of secretory cell duct.

The ducts of secretory cells showed FMRF amide-like IR in their sub-tegumental reservoirs situated near the apical pit of the scolex, where they occurred side-by-side with a large number of tubulin IR neurites of receptor cells (Fig. 5A and C). Terminal parts of the reservoirs were formed as thin projections, which were directed towards the tegument (Fig. 5C). The excretory system possessed a FMRF amide-like IR cortical network of excretory canals, which were associated with cell nuclei (Fig. 5B). These canals were connected with much smaller collecting ducts, whose funnel-like terminal enlargements surrounded tubulin IR flame flagellum of flame cells (Fig. 5D).

## Discussion

### Nervous system in the plerocercoids of diphyllobothriidean tapeworms

Species examined in this study shared the general distribution of FMRF amide-like IR in their nervous system; however, some differences were observed in the structure of their brain commissures. Both species possessed a single intensively FMRF amide-like IR commissural neuron, whose immunoreactivity markedly exceeded that of the others, but some variability was observed in an arrangement and a size of perikarya (soma) of these neurons. In *D. ditremus*, intensively FMRF amide-like IR perikaryon was situated posterior to those of other commissural neurons and its width was almost twice as width of weakly IR perikarya. Unlike its congener, *D. latus* had an intensively FMRF amide-like IR perikaryon situated more anteriorly, i.e. approximately in the middle of the brain commissure, and its soma was only slightly wider than that of the other commissural neurons. Nevertheless, commissural perikarya markedly exceeded dimensions of neuron somas associated with brain lobes in both species.

Structural differences in the brain commissure were also observed in other broad tapeworms elsewhere, and amidergic, peptidergic and serotonergic types of neurons were immunochemically differentiated (e.g. Gustafsson *et al*., 1985, 1986, 1994; Gustafsson, 1991; Gustafsson and Eriksson, 1992). For instance, Gustafsson (1991) observed only single RF amide-like IR large commissural neuron in *D. dendriticus*, while Biserova *et al*. (2020) summarized that the brain commissure of *D. dendriticus* possesses 13 neurons, among which 4 were (FM)RF amide-like IR.

There is not much comparable data for the model tapeworms, *D. ditremus* and *D. latus*. Biserova and Kemaeva (2012) described ultrastructure of 3 neuron somas in the commissure of *D. ditremus*. In *D. latus*, 2 perikarya (erroneously described as 2 commissures) occurred in the commissure, but their FMRF amide-like IR could not be confirmed due to application of a mixture of 3 neuromarkers (anti-FMRF amide, anti-serotonin and anti-synapsin antibodies) (Barčák *et al*., 2019).

As for other members of the order Diphyllobothriidea, Biserova and Gordeev (2010) found numerous commissural neurons (termed as giant neurons) in *Ligula intestinalis* (Linnaeus, 1758). Biserova *et al*. (2022) described these neurons in marine broad tapeworm *Pyramicocephalus phocarum* (Fabricius, 1780) and concluded that the brain commissure possessing large neuron somas might be characteristic for diphyllobothriidean species. The present data are congruent with this statement, and moreover, the current examination of *D. ditremus* suggests that intensively FMRF amide-like IR perikaryon in the brain commissure represents a multipolar neuron, i.e. the same type as was observed in *D. dendriticus* (Gustafsson, 1991).

No obvious differences were found between the 2 species described here, considering that the FMRF amide-like IR in 2 brain lobes are connected with main nerve cords, nerve plexuses and neurites of receptor cells, and that their architecture was similar to that of other broad tapeworms. Briefly, each brain lobe of *D. ditremus* (current study) and *D. dendriticus* (Gustafsson *et al*., 1985; Biserova and Kutyrev, 2014) split anteriorly into dorsal and ventral parts, and their neurites run either towards the tegument of inner and outer surface of bothria or to the apical part of the scolex. The main nerve cords exhibited the same pattern of FMRF amide-like IR as was repeatedly recorded in *D. dendriticus* (Gustafsson *et al*., 1985; Gustafsson, 1991; Biserova *et al*., 2014). Slightly different situation was reported in *P. phocarum* (Biserova *et al*., 2022), where both the FMRF amide-like IR neuron somas and their neurites occurred on the periphery of the main cords (and of the brain lobes, as well) rather than inside of these structures as observed in the current study and in previously published data on *D. dendriticus* (Gustafsson *et al*., 1985; Gustafsson, 1991; Biserova *et al*., 2014). In both diphyllobothriidean species examined in this study, FMRF amide-like IR nervous plexuses were situated close to the body surface (subtegumental plexus) and another plexus surrounded the main nerve cords. This FMRF amide-like IR network of neurites might serve as innervation of main longitudinal muscle bundles, because well-developed peptidergic plexuses occur near the musculature of flatworms (e.g. see review in Halton and Maule, 2004).

In *D. ditremus* and *D. latus* examined in this study, the receptor cells were recognized based on their sensilla embedded in the tegument and the neurites connected with the brain lobes or nearby nerve plexus in the scolex parenchyma. These tapeworms possessed sensilla distributed both in the apical area and on the bothrial surface, and those showing FMRF amide-like IR were much less numerous than tubulin IR sensilla. Morphologically, 3 types of the FMRF amide-like IR sensilla (fibre-like, bulb-like and flatten type), and a single type (bulb-like) of tubulin IR sensilla were observed in both species. However, the size of these bulb-like sensilla was different comparing *D. ditremus* and *D. latus*, as those of the latter were almost twice as wide as the sensilla of the former. Moreover, the tubulin IR bulb-like sensilla situated near the apical pit of *D. latus* differed in their internal structure, as some of them had an external cilium of different length, others contained an internal structure resembling the rootlet of microtriches, whereas the rest of the sensilla seemed to lack central tubulin IR structures.

Several types of sensilla distinguished by their ultrastructure (e.g. presence of the cilium and central rootlet) and their position in the tegument were recognized in diphyllobothriideans by transmission electron microscopy (e.g. Mustafina and Biserova, 2017). Biserova and Kemaeva (2012) recorded both ciliated and un-ciliated sensilla near the apical pit of *D. ditremus*, while Barčák *et al*. (2019) observed only un-ciliated sensilla with central rootlet situated near basal lamina of the tegument of *D. latus*. Higher variability of this structure was described in the scolex tegument of *D. dendriticus* by Kutyrev *et al*. (2017), who recognized 4 types of sensilla. Two ciliated types differed with each other by the length of the cilium, while un-ciliated types were distinguished based on the presence or absence of the central rootlet of microtubules. Similarity between these ultrastructural observations and the current data suggests that immunofluorescent techniques can also provide useful information about the system of sensilla on the diphyllobothriidean tapeworms, especially considering their spatial distribution.

Only few data are available on synapsin IR in tapeworms. The immunoreactivity of SYNORF1 (i.e. the same neuronal marker as used in the current study) was tested in cyclophyllidean tapeworms *Hymenolepis diminuta* (Rudolphi, 1819) by Rozario and Newmark (2015), who observed specific signal in both newly excysted juveniles and adults. The synapsin IR occurred in the brain, the neurites around the suckers and the rostellum, and posteriorly, it was captured in the longitudinal and transversal nerve cords and in a proximity of genital organs (Rozario and Newmark, 2015). Our observations on the plerocercoids of *D. ditremus* revealed synapsin IR in the brain lobes, main nerve cords and the neurites running towards the body surface. In the anterior part of main nerve cord, synapsin-IR occurred around the FMRF amide-like IR neurite, while posteriorly, the main nerve cords seemed to be thinner in diameter and both signals almost co-localize. In general, synapsin-IR exhibited similar distribution as FMRF amide-like IR, except the fact that the former was not observed in perikarya of our specimens. The current data represent the first known record of synapsin IR in diphyllobothriidean tapeworms.

### Secretory gland apparatus and excretory system

To date, the secretory cell ducts of *D. ditremus* and *D. latus* have been studied mainly by transmission electron microscopy (Andersen, 1975; Kuperman and Davydov, 1981; Biserova and Kemaeva, 2012; Barčák *et al*., 2019). The cytoplasm of these secretory cells contains well-developed granular endoplasmatic reticulum, which together with the presence of numerous mitochondria, ribosomes and secretory vesicles suggests active protein synthesis. The secretory cell bodies form a complex syncytial structure, which sends large dense vesicles (up to 1000 nm) through ducts reinforced by peripheral microtubules into subtegumental reservoirs near the apical pit of the scolex (Kuperman and Davydov, 1981). Finally, thin projections of the reservoirs penetrate the tegument and release the vesicles into an environment *via* eccrine secretion (Kuperman and Davydov, 1982). However, a chemical composition of these secretory vesicles is still poorly known in tapeworms, because granules were mostly unreactive to cytochemical tests (Öhman-James, 1973). A clue may be provided by the immunogold labelling of tapeworm nervous system, because similar large dense vesicles (>140 nm) in the neurites of flatworms were FaRPs IR (Reuter and Gustafsson, 1989; Brennan *et al*., 1993; Halton *et al*., 1994). Therefore, the large dense vesicles in the ducts of the secretory cells, which share similar ultrastructure with FaRPs IR vesicles in the nervous system, could also be amidergic in nature. This assumption is supported by the present data based on an examination of whole-mount specimens, because FMRF amide-like IR was observed in the ducts of secretory cells, especially in their subtegumental reservoirs. The ducts originated from the posterior part of the scolex, ran laterally to the brain towards the apical part of the scolex and opened into and near to the apical pit, where large reservoirs were formed under the tegument. The FMRF amide-like IR also occurs in the apical pit itself, which may possibly represent an aggregation of amidergic secretory products of these cells. The current data on *D. ditremus* and *D. latus* thus update the knowledge on the spatial distribution of the secretory gland apparatus (i.e. frontal glands) and the chemical nature of its secretion in 2 broad tapeworm species.

Most of immunohistochemical examinations of tapeworms have focused on their nervous system, while the excretory system has remained fairly neglected. A rare exception is the work of Rozario and Newmark (2015), who labelled the excretory system of adult *H. diminuta* by several antibodies and lectins (an anti-FaRP marker was not used), and depicted its microanatomy from flame cells to main excretory canals. Considering diphyllobothriideans, most of the available data were obtained on the sectioned specimens of both adult and plerocercoids of *D. dendriticus*, in which immunoreactivity against histidine–isoleucin peptide, gastrin, NADPH diaphorase and collagen were captured in or near the wall of main excretory canals (Gustafsson *et al*., 1986; Lindroos and Still, 1990; Lindholm *et al*., 1998). Among other markers, anti-FMRF amide antibodies were used to label both adult and plerocercoids of *D. dendriticus*, but the specific signal in the excretory system was not observed (Gustafsson *et al*., 1985, 1986). In the recent work of Biserova *et al*. (2021) the network of excretory canals in plerocercoids of *P. phocarum* was described as a syncytial structure with cell nuclei associated with the canal walls, similarly as was observed in this work. In *D. ditremus* and *D. latus* characterized herein, the FMRF amide-like IR occurred in well-developed network of excretory canals in cortical parenchyma as well as in collecting ducts associated with flame cells. However, these observations did not allow us to decide whether the immunoreactivity is caused by structural peptides in the wall of the excretory system or by ‘waste’ molecules, which are going to be released into the tapeworm's environment.

### A cross-reactivity of anti-FMRF amide antibodies and their target (neuro)peptide antigens in *Dibothriocephalus* spp.

The anti-FMRF amide antibodies obviously cross-react in flatworms, because the original FMRF amide has never been confirmed in these invertebrates. The target antigens for these antibodies are some molecules structurally similar to this mollusc tetrapeptide, most probably those with –RF amide-terminated carboxy end (Lange *et al*., 1991). This amino acid residue thus seems to be the specific epitope responsible for FMRF amide-like IR in flatworms. As knowledge about structural variability of these amidated peptides is limited in tapeworms, their primary structure in diphyllobothriideans could only be estimated based on their amino acid sequences known in other tapeworms, or predicted from available genomic and transcriptomic data.

To date, only 2 peptides with –RF amide terminal were exactly determined in tapeworms, both in cyclophyllidean *Moniezia expansa* (Rudolphi, 1810). The first of them, the hexapeptide GNFFRF amide, belongs to the FaRPs peptide family and the second, 39-amino acid residue peptide terminated with –FAIIGRPRF amide sequence is related to neuropeptide F (NPF) family (Maule *et al*., 1991, 1993*b*). These 2 peptides were immunocytochemically confirmed as the antigens for anti-FMRF amide antibodies (Maule *et al*., 1993*a*, 1993*b*), and therefore, their structural analogues in other tapeworms most probably also cross-react in a similar way. Additionally, 3 possible precursors of NPFs, which coded –GR[P/A]R[F/Y]amide-terminated peptides, were predicted in *M. expansa* (McVeigh *et al*., 2009).

Although no –RF amide-terminated peptide has been directly isolated from any broad tapeworm, 3 gene precursors of such peptides were predicted in the genome of *D. latus*. The first of them coded GNIFRF amide, which possessed 1 amino acid substitution (GN[I/F]FRF amide) compared with FaRP of *M. expansa*; the second one coded 40-residue NPF terminated with –FALIGRPRF amide, which also differs only by a single substitution (–FA[L/I]IGRPRFamide) regarding terminal nonapeptide of the cyclophyllidean NPF; the third precursor coded 17-residue-long peptide (–WRPHSRF amide) from Luqin family, another group of invertebrate neuropeptides (Shyamala *et al*., 1986; Koziol *et al*., 2016). Structural similarities between anti-FMRF antibody-binding peptides in *M. expansa* and –RF amide-terminated peptides predicted in *D. latus* genome suggested that the latter might be responsible for FMRF amide-like IR observed in our specimens.

The FMRF amide-like IR of the nervous system in our specimens of *D. ditremus* and *D. latus* corresponded with the fact that all of above-mentioned peptides are known as neuroactive substances (Shyamala *et al*., 1986; Maule *et al*., 1991, 1993*b*). However, the secretory gland apparatus and the excretory system of both *D. ditremus* and *D. latus* also exhibited FMRF amide-like IR, which can be attributed to either (i) an occurrence of above-mentioned (neuro)peptides in the secretory and excretory systems or (ii) synthesis/release of other, currently unknown short peptides, which are probably ended with –RF amide carboxy terminal. Future application of specific antibody markers synthesized against predicted peptides could elucidate the distribution of these molecules in tapeworms and help to understand the complexity of their nervous system and secretory/excretory apparatus.

## Data Availability

The authors confirm that the data supporting the findings of this study are available within the article and its supplementary materials.

## Supplementary material

For supplementary material accompanying this paper visit https://doi.org/10.1017/S0031182023000306. click here to view supplementary material
