# Supplementary figures and images for: An examination of nervous system revealed unexpected immunoreactivity of both secretory apparatus and excretory canals in plerocercoids of two broad tapeworms (Cestoda: Diphyllobothriidea)
Source: Parasitology. 2023 Mar 20;150(7):612–22. doi: 10.1017/S0031182023000306 (PMC10260320; doi:10.1017/S0031182023000306)

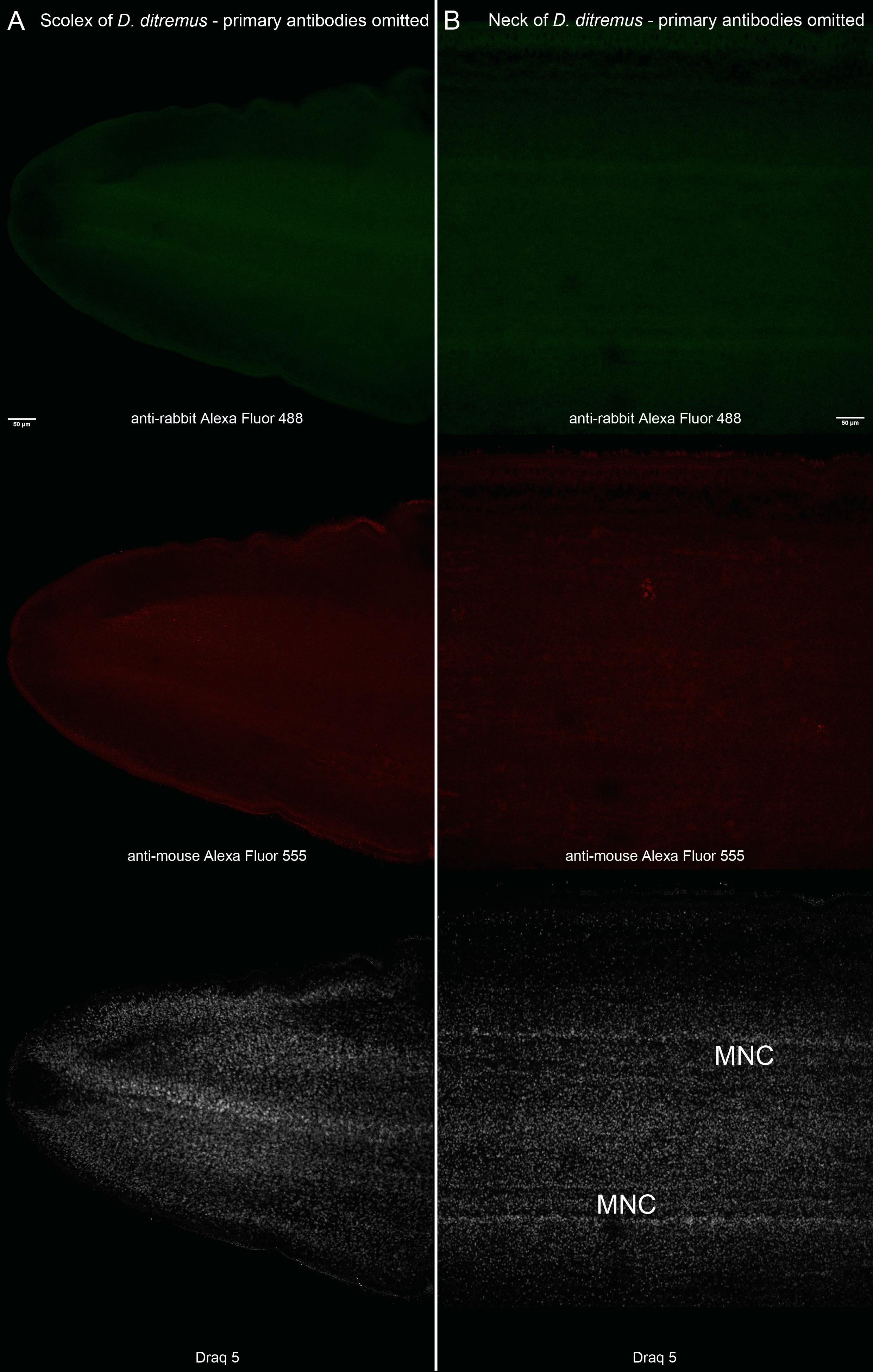

Supplement: Supplementary file 1 [file S0031182023000306sup.zip › S0031182023000306sup001.tif]

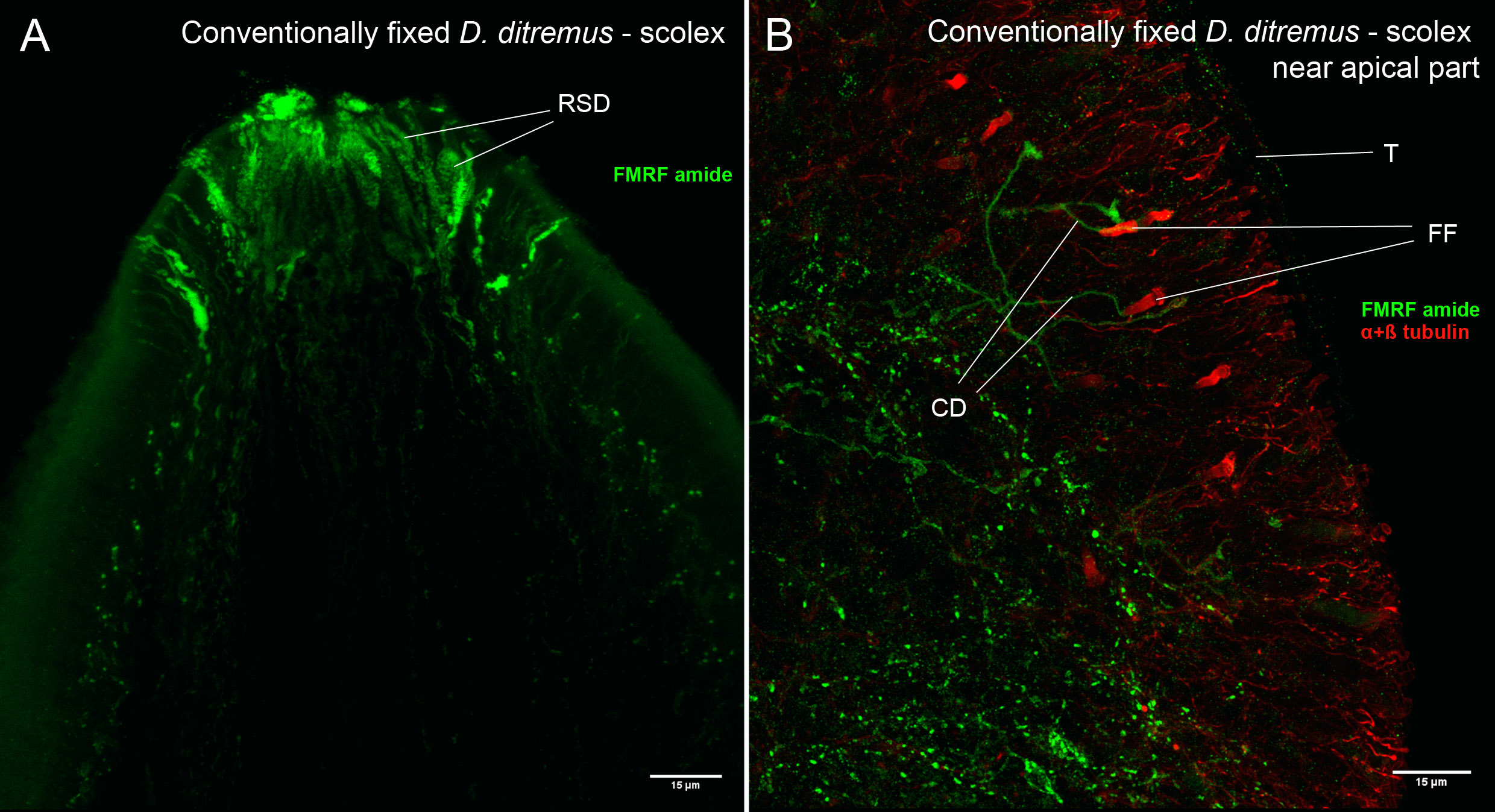

Supplement: Supplementary file 1 [file S0031182023000306sup.zip › S0031182023000306sup002.tif]
